# Hepatic failure, neonatal hemochromatosis and porto-pulmonary hypertension in a newborn with trisomy 21 - a case report

**DOI:** 10.1186/1824-7288-36-38

**Published:** 2010-05-18

**Authors:** Erin Neil, Josef Cortez, Aparna Joshi, Erawati V Bawle, Janet Poulik, Mark Zilberman, Mohammad F El-Baba, Beena G Sood

**Affiliations:** 1 Department of Pediatrics, Children's Hospital of Michigan, 3901 Beaubien St., Detroit, MI, 48201 USA; 2 Division of Neonatal-Perinatal Medicine, Children's Hospital of Michigan, 3901 Beaubien Blvd., Detroit, MI, 48201 USA; 3 Department of Pediatric Radiology, Children's Hospital of Michigan, 3901 Beaubien Blvd., Detroit, MI, 48201 USA; 4 Division of Genetic and Metabolic Disorders, Children's Hospital of Michigan, 3901 Beaubien Blvd., Detroit, MI, 48201 USA; 5 Department of Pediatric Pathology, Children's Hospital of Michigan, 3901 Beaubien Blvd., Detroit, MI, 48201 USA; 6 Division of Pediatric Cardiology, Children's Hospital of Michigan, 3901 Beaubien Blvd., Detroit, MI, 48201 USA; 7 Division of Pediatric Gastroenterology; Children's Hospital of Michigan, 3901 Beaubien Blvd., Detroit, MI, 48201 USA

## Abstract

Liver failure in neonates is a rare but often fatal disease. Trisomy 21 is not usually associated with significant infantile liver disease. If present, hepatic dysfunction in an infant with Trisomy 21 is likely to be attributed to transient myeloproliferative disorder with hepatic infiltration by hematopoietic elements and may be associated with secondary hemosiderosis. A less commonly recognized cause of liver failure in neonates with Trisomy 21 is neonatal hemochromatosis (NH); this association has been reported in nine cases of Trisomy 21 in literature. NH is a rare, severe liver disease of intra-uterine onset that is characterized by neonatal liver failure and hepatic and extrahepatic iron accumulation that spares the reticuloendothelial system. NH is the most frequently recognized cause of liver failure in neonates and the commonest indication for neonatal liver transplantation. Although porto-pulmonary hypertension (PPH) has been reported as a complication of liver failure in adults and older children, this has not been reported in neonates with liver failure of any etiology. This is probably due to the rarity of liver failure in newborns, delayed diagnosis and high mortality. The importance of recognizing PPH is that it is reversible with liver transplantation but at the same time increases the risk of post-operative mortality. Therefore, early diagnosis of PPH is critical so that early intervention can improve the chances of successful liver transplantation. We report for the first time the association of liver failure with porto-pulmonary hypertension secondary to NH in an infant with Trisomy 21.

## Background

Neonatal hemochromatosis (NH) is the most frequently recognized cause of liver failure in neonates and the commonest indication for neonatal liver transplantation. Porto-pulmonary hypertension, a serious complication of liver failure in adults and children, has not previously been described in neonates. Trisomy 21 is not usually associated with significant infantile liver disease [1]. Hepatic dysfunction in an infant with Trisomy 21 is likely to be attributed to transient myeloproliferative disorder (TMD) with hepatic infiltration by hematopoietic elements and may be associated with secondary hemosiderosis [2]. A less commonly recognized cause of liver failure in neonates with Trisomy 21 is NH; this association has been reported in nine cases of Trisomy 21 in literature [3]. We report for the first time the association of porto-pulmonary hypertension with liver failure secondary to NH in an infant with Trisomy 21.

## Case Presentation

BGB was delivered at 37 weeks (birth weight 3550 g) gestation by a 37 year old Caucasian woman with a past history of one term delivery, one ectopic pregnancy and one miscarriage. She declined prenatal serum triple screen testing. Cesarean section was performed for non-reactive non-stress test. Apgar scores were 7 and 8 at one and five minutes respectively. The infant was noted to have a vigorous cry, cyanosis, generalized edema, diffuse ecchymoses and petechiae, and physical features suggestive of Down syndrome; the latter diagnosis was subsequently confirmed on chromosomal analysis which showed Trisomy 21. Oxygen saturation in room air was 59% and improved with supplemental oxygen by hood. Initial white cell count was 20,700/cmm, hematocrit 38% and platelet count 37,000/cmm. A chest radiograph revealed cardiomegaly. At one hour of age the infant was noted to have a loud holosystolic murmur over the precordium. Early persistent hypoglycemia despite treatment was documented. The infant was transferred to a level III NICU for evaluation for a heart defect and non-immune hydrops in an infant with probable trisomy 21. The placental pathology was later reported as hydropic placenta with villous dysmaturity, pigment laden macrophages, and microcalcifications with no evidence of villitis or leukemic infiltrates.

**Figure 1:**
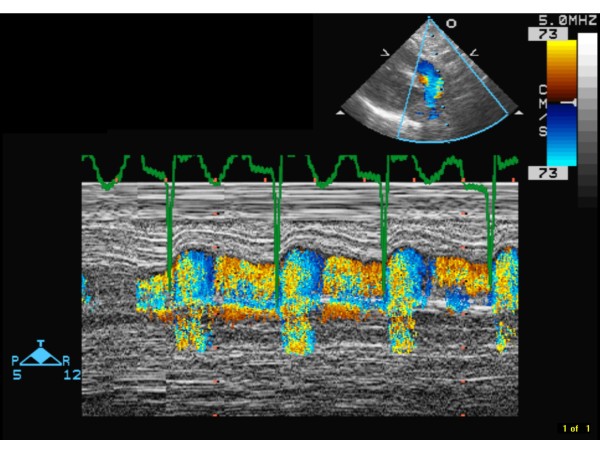
**Color M-mode of ductal flow on echocardiography**. In systole (as indicated by the ECG marker) the flow is blue, i.e. from the pulmonary artery into the aorta; in diastole (as indicated by the ECG marker) the flow is red, i.e. from the aorta into the pulmonary artery.

**Figure 2:**
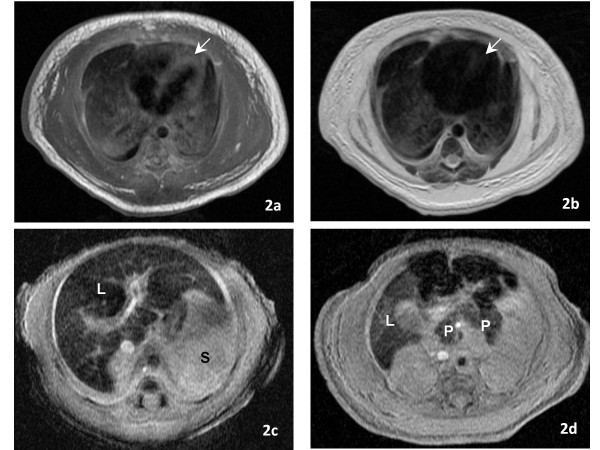
**a and b: T1-weighted (1A) and T2-weighted (1B) spin echo axial MR images at the level of the heart show marked drop in myocardial signal intensity (arrow) on T2-weighted image, reflecting myocardial hemosiderin deposition**. c and d: Axial T2 STAR gradient echo MR images of the abdomen at the level of the liver and spleen (Fig. 2) and liver and pancreas (Fig. 3) show low signal intensity in the liver (L) and pancreas (P) and normal signal intensity in the spleen (S), reflecting hepatic and pancreatic siderosis.

**Figure 3:**
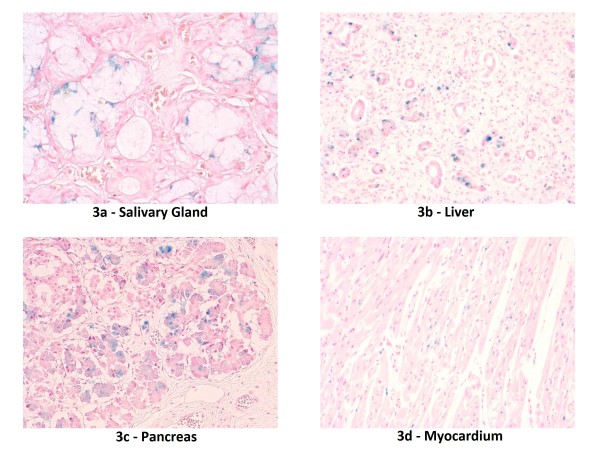
**Prussian blue stain showing iron deposition (original magnification × 200)**. a - Salivary gland with iron deposition in glandular epithelium. b - Liver with diffuse fibrosis, ductular proliferation, fibrous obliteration of central veins and hepatocellular and ductular iron deposition. c - Pancreas with interstitial fibrosis and deposition of iron in acinar epithelial cells. d - Myocardium with perinuclear iron deposition.

At the referral hospital, an echocardiogram showed normal anatomy with evidence of pulmonary hypertension. Direct hyperbilirubinemia was observed on the first day of life (D1) (total bilirubin 10.4, direct bilirubin 3.6 mg/dl). Coagulation studies were abnormal. TMD was suspected; however, flow cytometry, peripheral smear and immunophenotyping were not supportive of the diagnosis. Abdominal ultrasound showed ascites, normal appearing liver, spleen and kidneys, and absence of portal hypertension. On D2, the infant developed hypotension that was treated with escalating doses of dopamine, dobutamine, and epinephrine drips and hydrocortisone. Her liver function progressively deteriorated, evident by worsening coagulation profile, increasing direct hyperbilirubinemia, hypoalbuminemia, hypoglycemia, and worsening edema. Laboratory test results included: normal serum transaminases, ammonia and lactate; serum albumin 1.1 g/dl; AFP 459.3 ng/ml; serum Fe 123 z-mcg/dl; TIBC 160 z-mcg/dl; Ferritin 525 ng/ml; Factor V activity 11%; Factor VII activity 3.2%; factor VIII activity 95%. Microbiologic and serologic work up ruled out TORCH, parvovirus, adenovirus and enterovirus infections. Urine reducing substances, quantitative plasma amino acids, and tests for organic acidemias, fatty oxidation defects and inborn errors of bile acid synthesis were normal. She was supported with blood, plasma, and albumin transfusions. On D3, she was placed on assisted ventilation for persistent hypoxemia. Inhaled nitric oxide was initiated on D7 for persistent pulmonary hypertension on echocardiogram (bidirectional shunting across foramen ovale and ductus arteriousus, tricuspid valve insufficiency, dilated right ventricle with markedly elevated right ventricular systolic pressure of 74 mm Hg; Figure 1) with only marginal improvement in oxygenation. A diagnosis of hepatic failure secondary to NH associated with porto-pulmonary hypertension was entertained. Magnetic resonance imaging of the abdomen was suggestive of NH (Figure 2). A lip biopsy on D8 confirmed hemosiderin deposition in salivary glandular epithelium (Figure 3a).

Treatment with double volume exchange transfusion followed by administration of immunoglobulin (IVIG) and an antioxidant cocktail of selenium, prostaglandin E_1_, N-acetyl-cyteine, desferoxamine, and vitamin E was initiated on D7. However, her cardiorespiratory status continued to deteriorate and she expired on the 10^th ^day of life. Permission for autopsy was obtained.

Microscopically, the liver showed diffuse fibrosis, ductular and pseudoductular proliferation, fibrous obliteration of central veins, and hepatocellular and cholangiolar cholestasis. Foci of extramedullary hematopoiesis were identified. No leukemic infiltrates were identified. Hepatocellular and ductular deposition of iron was confirmed (Figure 3b). The pancreas showed diffuse fibrosis without megakaryocytes or leukemic infiltrates. Moderate iron deposition was demonstrated in the pancreas and myocardium but not in the spleen (Figure 3c-d). Microscopic examination of the lungs revealed interstitial fibrosis, focal type II pneumocyte hyperplasia, and few peripheral arterioles with thick walls. The radial alveolar count was less than three in many areas (expected 4-5).

## Discussion

Liver failure in neonates is a rare but often fatal event. Causes include inborn errors of metabolism, perinatal infections, hypotension/shock, and hematological conditions like TMD, congenital leukemia and hemophagocytic lymphohistiocytosis [4,5]. Whereas metabolic liver diseases typically present weeks to months after birth and infectious diseases weeks after birth, neonatal NH presents soon after birth. In NH, the onset of the liver disease is in utero, with end-stage liver disease already established even in the prematurely born infant [6,7]. It is the most frequently recognized cause of liver failure in neonates and the commonest indication for neonatal liver transplantation.

In NH, severe fetal liver injury often leads to fetal loss as evidenced by the obstetric histories of women who have had an infant diagnosed with NH [7,8]. Intrauterine growth restriction, oligohydramnios or polyhydramnios, fetal distress and placental edema may be present. Infants who are not stillborn exhibit liver and multiorgan failure within the first few days of life. Jaundice with significant elevations of both conjugated and nonconjugated bilirubin, hypoglycemia, marked coagulopathy, thrombocytopenia, anemia, hypoalbuminemia, edema, ascites, and oliguria are prominent features [6]. Typical biochemical findings include an extremely high serum ferritin level (usually > 800 ng/mL), and extremely high levels of AFP (usually > 200 ng/mL). An unusual characteristic given the degree of liver injury is low or absent serum transaminases. Hepatocellular synthetic insufficiency leads to hypoglycemia, coagulopathy (factors V and VII usually less than 10% of normal, low fibrinogen), hypoalbuminemia (usually less than 2 gm/dL) and decreased iron-binding capacity. Liver histology is characterized by intense fibrosis and cirrhosis without acute necrosis. However, none of these findings is diagnostic of NH.

The diagnosis of NH is based on the clinical features of liver failure at birth with extrahepatic siderosis and sparing of the reticuloendothelial system in the absence of other causes of liver failure. Extrahepatic siderosis can be demonstrated non-invasively in these critically ill infants on magnetic resonance imaging (MRl) and lip biopsy [6,9-11]. Hepatic siderosis is not specific for NH, rather it is observed in the normal infant liver and more prominently in a multitude of liver diseases. Therefore liver biopsy, a hazardous procedure in the presence of coagulopathy, is not diagnostic for NH.

The prognosis for patients with NH is grim; most succumb to the complications of end-stage liver disease within the first few weeks of life if not successfully rescued by liver transplantation [7,12-16]. Treatment with either anti-oxidant/chelator cocktail, or exchange transfusion and IVIG has been reported to be successful in small series of patients but these therapies have not been systematically evaluated. The survival after liver transplantation is 50% (median follow up 7.8 years, range 3-10 years). There is a high recurrence rate (~80%) of NH in families following the birth of an affected child.

The case presented here had several typical features of NH. The unique features of the infant presented in this report are the association of NH with Trisomy 21 (tenth reported case in literature) and the association of NH with PPH. Although PPH has been reported with end-stage liver disease (ESLD) in adults and older children, this is the first report of PPH in association with liver failure secondary to NH, an end-stage liver disease seen in the neonatal period [17]. The diagnosis of PPH in adults and older children requires the presence of pulmonary hypertension in the setting of liver disease or portal hypertension. It has been proposed that binding of endothelin-A receptors by increased circulating levels of endothelin 1 (ET-1) produced by the cirrhotic liver lead to vasoconstriction and vascular smooth muscle proliferation which manifests as PPH. Although the diagnosis of PPH is made on the basis of hemodynamic criteria, presence of morphologic features of pulmonary hypertension has been described in a few case series of pediatric ESLD [18,19]. Because of the rarity of PPH, diagnosis is often delayed. Clinical diagnosis requires a high degree of suspicion and deliberate follow-up by echocardiography for the timely detection of pulmonary hypertension before irreversible vascular damage occurs. In our patient, pulmonary hypertension that was present since the first day of life worsened progressively inspite of treatment. Infants with Trisomy 21 have an increased risk for developing persistent pulmonary hypertension in the neonatal period as well as primary pulmonary hypertension later in life even in the absence of structural heart disease [20,21]. The predisposition to neonatal pulmonary hypertension has been attributed to a combination of reduced alveolar count (as was observed on autopsy in our patient), reduced capillary surface area and an abnormal pulmonary vasculature. Pulmonary hypertension in neonates with Trisomy 21 typically responds to medical therapy. Several features suggest that the pulmonary hypertension observed in this neonate was attributable to ESLD, with the presence of Trisomy 21 being a contributory factor - the fact that the hypoxemia responded to supplemental oxygen in the first two days and then worsened progressively with worsening liver failure despite treatment with assisted ventilation and inhaled nitric oxide. The neonate with ESLD differs from adults and children with ESLD in that pulmonary hypertension is normal during fetal life because the placenta, not the lung, serves as the organ of gas exchange and the presence of ESLD may prevent the normal postnatal drop in pulmonary vascular resistance soon after birth. Therefore hemodynamic criteria for porto-pulmonary hypertension may be present in the absence of morphologic criteria because of presentation of liver failure soon after birth. The importance of recognizing PPH is that it is reversible with liver transplantation but at the same time increases the risk of post-operative mortality. It has been suggested that screening with echocardiography be considered for all adults and children with ESLD for early diagnosis of PPH. We suggest that this recommendation be also applied to newborns with fulminant liver failure to improve outcomes.

## Conclusions

This case emphasizes the importance of recognition of NH as a cause of liver failure in infants with Trisomy 21 and the association porto-pulmonary hypertension with neonatal liver failure. The finding of coagulopathy and hyperbilirubinemia in an infant with hypoalbuminemia, ascites, or splenomegaly should prompt the consideration of neonatal liver failure. Although NH is a rare disease, it is the commonest cause of neonatal liver failure and should be part of the differential diagnoses of any neonate presenting with liver failure. Lip biopsy and MRI to demonstrate extrahepatic siderosis are important in confirming the diagnosis after excluding other causes. These infants should be screened for PPH by echocardiography as this influences outcome. Early recognition of NH can improve survival by early referral for liver transplantation. Diagnosis of NH also has implications for recurrence in future pregnancies.

## Consent

Written informed consent was obtained from the patient's parents for publication of this case report and accompanying images. A copy of the written consent is available for review by the Editor-in-Chief of this journal.

## Competing interests

The authors declare that they have no competing interests.

## Authors' contributions

All authors participated in the care of the infant and have read and approved the final manuscript.
